# Non-Surgical Treatments of Trigeminal Neuralgia from the Perspective of a Pain Physician: A Narrative Review

**DOI:** 10.3390/biomedicines11082315

**Published:** 2023-08-21

**Authors:** Jin Young Lee, Gil Ho Lee, Seung Hyun Yi, Woo Seog Sim, Bae Wook Kim, Hue Jung Park

**Affiliations:** 1 Department of Anesthesiology and Pain Medicine, Samsung Medical Center, School of Medicine, Sungkyunkwan University, Seoul 06351, Republic of Korea; l7035@hanmail.net (J.Y.L.);; 2 Department of Anesthesiology and Pain Medicine, Seoul St. Mary’s Hospital, College of Medicine, The Catholic University of Korea, Seoul 06591, Republic of Korea

**Keywords:** intervention, pain, pharmacotherapy, treatment, trigeminal neuralgia

## Abstract

Trigeminal neuralgia (TN) is a unilateral disorder characterized by electric shock-like pain, abrupt onset and termination, and limited to one or more branches of the trigeminal nerve. Various therapeutic modalities for TN have been introduced. We searched for literature indexed in PubMed, Medline, and the National Library of Medicine and reviewed all relevant articles on non-surgical treatments for TN. Published studies were reviewed with no restrictions on date; reviews, clinical trials, animal studies, retrospective studies, and cases were included. Carbamazepine and oxcarbazepine are the recommended first-line pharmacotherapies. Interventional treatments should be considered when pharmacotherapy is insufficient or withdrawn because of adverse effects.

## 1. Introduction

Trigeminal neuralgia (TN) is characterized by sudden shock-like pain, abrupt onset and termination, and is limited in distribution to one or more divisions of the trigeminal nerve [1]. The subtypes of TN are defined by the International Headache Society with the International Classification of Headache Disorders Edition 3 (ICHD-3) as classical (75%), idiopathic (10%), and secondary (15%) [2,3]. Classical TN is caused by the compression of abnormally twisted blood vessels, such as the superior cerebellar and basilar arteries [2,4]. Vascular compression of the trigeminal root in the entry zone of the pons causes demyelination and neuronal loss. These changes lower the excitability threshold of the affected fibers and promote inappropriate firing toward adjacent fibers, which causes the signs and symptoms of TN [2,3]. Idiopathic TN is defined when neither electrophysiological tests nor MRI show significant abnormalities [2]. Classical and idiopathic TN are subdivided into paroxysmal and concomitant continuous pain types [2]. In the concomitant continuous pain type, the trigeminal nerve root is more severely atrophic than in the paroxysmal type, indicating that continuous pain is most likely related to axonal loss and abnormal activity in the trigeminal neurons [3]. Secondary TN is caused by neurological diseases, including inflammatory/demyelinating diseases (multiple sclerosis and sarcoidosis), tumors, other vascular lesions, connective tissue disorders, congenital diseases, and other systemic conditions affecting the trigeminal nerve [5]. Despite recent advances in TN treatment, evidence for the proper management of pain remains insufficient. Non-surgical options are based on pharmacotherapy and interventional treatments [6,7,8,9,10]. Sodium channel blockers have shown significant clinical effects, suggesting that TN is a form of sodium channelopathy. In particular, the voltage-gated sodium channels, Nav1.7, Nav1.3, and Nav1.8 are abnormally expressed in the TN and cause rapid activation and inactivation, as well as maintenance of the action potential [3]. Interventions include non-ablative percutaneous procedures (nerve block and botulinum toxin injection), ablative percutaneous procedures (chemodenervation, radiofrequency ablation, and balloon compression), and neuromodulation (transcutaneous electrical nerve stimulation, direct nerve stimulation, deep brain stimulation, motor cortex stimulation, and transcranial MR cortical stimulation) [5]. Here, we reviewed the efficacy and safety of the non-surgical treatments for TN.

## 2. Methods

### 2.1. Protocols

**Figure 1:**
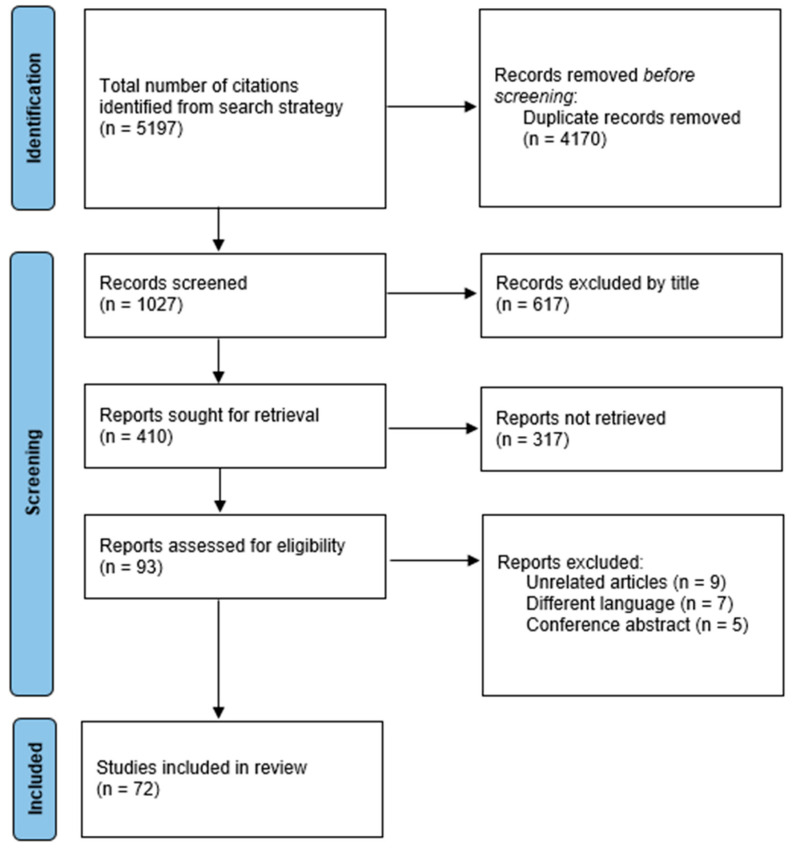
Overview of the systematic review process.

This narrative review applied the guidelines issued in the latest Preferred Reporting Items for Systematic Reviews and Meta-Analysis (Figure 1).

### 2.2. Information Sources

The PubMed, Medline, and National Library of Medicine databases were searched for literature, and all relevant articles on TN treatment were reviewed. The Medical Subject Heading (MeSH) terms “trigeminal neuralgia” and “pharmacotherapy” or “interventions” or “therapies” were included in the search. Published studies were reviewed with no restriction on date, and reviews, clinical trials, animal studies, retrospective studies, and cases were included. Only studies in which the complete text was available in English were included.

## 3. Pharmacologic Treatments

Pharmacotherapy with anti-epileptics is the standard treatment for TN [5,11,12] (Table A1, Appendix A). Secondary TN should be treated for underlying pathologies. Before pharmacotherapy, baseline and follow-up electrocardiograms, electrolytes, and hematologic tests should be performed to ensure adequate heart, liver, and renal function [13]. Membrane stabilizing agents, such as anti-epileptics, control lancinating or paroxysmal pain by suppressing ectopic transmission by blocking sodium channels [12,14]. Carbamazepine and oxcarbazepine are the first-line pharmacotherapies [15]. The second line includes lamotrigine and baclofen [16]. The third line includes gabapentin, pregabalin, and topiramate [17]. The second and third lines of therapy can be used either alone or as add-on therapies [3]. Carbamazepine and oxcarbazepine produce a frequency-dependent block of voltage-gated sodium channels by reducing the frequency of action firing, which is effective against paroxysmal pain [18]. However, they had a lesser positive effect on concomitant persistent pain [18]. Gabapentinoids and antidepressants might be effective in treating persistent pain and are recommended as add-ons to first-line drugs in patients with atypical TN with concomitant persistent pain [18]. The American Academy of Neurology and the European Federation of Neurological Societies established clinical guidelines for TN, with carbamazepine as effective, oxcarbazepine as probably effective, and lamotrigine and baclofen as possibly effective in controlling pain in classic TN [19]. When pharmacotherapy is insufficient or withdrawn because of adverse effects, interventional treatments should be considered.

### 3.1. Carbamazepine and Oxcarbazepine

#### 3.1.1. Carbamazepine

**Figure 2:**
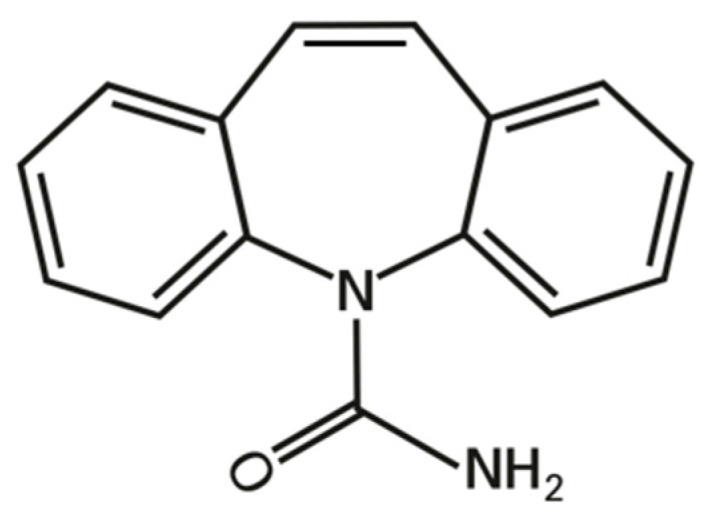
Carbamazepine.

Carbamazepine is used to treat various types of neuropathic pain. It acts by inhibiting voltage-gated sodium channels, thereby reducing the excitability of the neural membrane [17]. Carbamazepine is an agonist of the alpha 1, beta 2, and gamma 2 subunits of gamma-aminobutyric acid (GABA) receptors [17] (Figure 2). It reduces the frequency and intensity of painful paroxysms and is equally effective against spontaneous and trigger-evoked attacks [19]. The initial dose is 100–200 mg twice daily and can be increased to 100 mg every second day until sufficient pain relief is attained with tolerable adverse effects. The typical maintenance dose is 300–800 mg daily in two to three divided doses. The maximum dose is 1200–1800 mg daily [17]. Common adverse effects include sedation, dizziness, nausea, vomiting, diplopia, ataxia, elevated hepatic enzyme levels, and hyponatremia [17]. Uncommon serious adverse effects include hematological reactions (leukopenia, aplastic anemia, and hepatotoxicity) and cutaneous problems (allergic rash, systemic lupus erythematosus, Stevens–Johnson syndrome, and toxic epidermal necrolysis), which cause withdrawal in up to 40% of patients [3,13,17]. Stevens–Johnson syndrome is a serious reaction of the skin and mucous membrane, which starts with fever, fatigue, and sore throat, followed by a painful skin rash, blisters, and peeling. Complications include sepsis, pneumonia, and multiple organ failure [17]. A strong genetic association between HLA-B^*^1502 and carbamazepine-induced Stevens–Johnson syndrome and toxic epidermal necrolysis have been reported in Han Chinese, Indian, Thai, and Asian populations [13]. The frequency of this allele is 5–10% in Asian populations [13]. Therefore, HLA-B^*^1502 testing is recommended to assess risk before starting carbamazepine [3,20]. Carbamazepine interacts metabolically with other medications, which can be problematic for patients with polypharmacy. The interaction between carbamazepine and warfarin results in decreased warfarin efficacy. Therefore, carbamazepine initiation should be accompanied by close coagulation monitoring to avoid thrombosis and ischemic stroke [21]. Regarding the efficacy of carbamazepine for TN treatment, the number needed to treat (NNT) to obtain pain relief is 1.7–1.8, and the number needed to harm (NNH) is 3.4 for minor and 24 for severe adverse events [11,19].

#### 3.1.2. Oxcarbazepine

**Figure 3:**
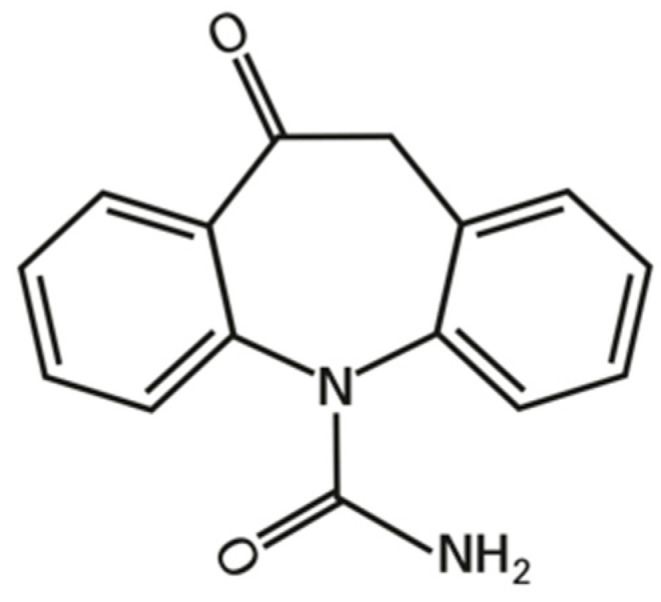
Oxcarbazepine.

Oxcarbazepine is a keto analog of carbamazepine that acts as a sodium channel modulator and has comparable efficacy to carbamazepine for TN [15] (Figure 3). Oxcarbazepine reduces the activity of high-voltage-activated potassium and calcium channels [22]. The starting dose of oxcarbazepine is 150 mg twice daily, which is increased to 300 mg every third day until pain relief is achieved. The maintenance dose is 300–600 mg twice daily, up to a maximum dose of 1200–2400 mg daily [17]. Oxcarbazepine has greater tolerability than carbamazepine, with a lower risk of allergic reactions and drug–drug interactions [15]. The dose change from carbamazepine to oxcarbazepine is carbamazepine 200 to oxcarbazepine 300 mg [3]. The adverse effects include excessive central nervous system (CNS) depression and dose-related hyponatremia [3]. Oxcarbazepine should be avoided in patients expressing the HLA-B^*^1502 allele due to the potential for rare skin reactions [15].

### 3.2. Lamotrigine and Baclofen

#### 3.2.1. Lamotrigine

Lamotrigine has a bimodal action mechanism that inhibits the release of the excitatory neurotransmitter glutamate through the inhibition of voltage-gated sodium channels and acts as an antagonist of N-methyl-D-aspartate (NMDA) receptors [16]. It is administered at 25 mg daily for the first two weeks and then increased to 50 mg daily for three to four weeks until a total daily dose of 100–400 mg, up to a maximum of 600 mg [16]. Common adverse effects include sleepiness, dizziness, headache, vertigo, and ataxia [17]. Stevens–Johnson syndrome can occur in one in 10,000 patients [17]. This can be prevented by a slower titration of the dose.

#### 3.2.2. Baclofen

Baclofen is approved by the Food and Drug Administration for the treatment of reversible spasticity related to multiple sclerosis, spinal cord injuries, and other spinal cord pathologies [23]. It has been used off-label for musculoskeletal pain, persistent or chronic hiccups, lower back pain, and TN [23]. Baclofen is an agonist of the beta subunit of GABA receptors in the pre- and post-synaptic neurons of the spinal cord and brain [23,24]. It inhibits the transmission of both mono and polysynaptic reflexes in the spinal cord, relaxes spasticity, and reduces substance P levels in the spinal cord [23]. Baclofen can be administered orally or intrathecally. For muscle spasms or musculoskeletal pain, oral baclofen starts at an initial dose of 5–10 mg, one to three times daily, with a maximum dose of 60–80 mg daily [23]. Baclofen reduces the number of painful episodes and prolongs remission. Its adverse effects include drowsiness, muscle weakness, fatigue, cognitive deficits, tolerance, and potential abuse [25]. If baclofen is combined with carbamazepine, the carbamazepine dose must be reduced to 500 mg daily to maintain the synergistic effects [17]. Due to the narrow therapeutic window of baclofen, careful monitoring of dose initiation and tapering is recommended [23]. Abrupt withdrawal is avoided due to life-threatening complications (e.g., pruritus, hyperthermia, multisystem organ failure, hyperreflexia, hallucinations, headache, delirium, and seizures) [11,23].

### 3.3. Pimozide and Tizanidine

Pimozide, a dopamine receptor antagonist, is used to manage Tourette syndrome [17]. In randomized, double-blind, crossover trials of 48 patients with refractory TN, pimozide was shown to be more effective than carbamazepine; however, side effects, including CNS disturbances, hand tremors, and memory impairment, have been reported [11,26]. However, in a network meta-analysis of patients with TN, pimozide did not show better efficacy than a placebo [27]. Tizanidine, a centrally acting alpha-adrenergic agonist, was compared with carbamazepine in six patients in each group, with maximal daily doses of 18 and 900 mg, respectively, and the effects of tizanidine were inferior to those of carbamazepine [28].

### 3.4. Gabapentin and Pregabalin

#### 3.4.1. Gabapentin

Gabapentin is a GABA receptor agonist that acts on pre-synaptic calcium channels of neurons to inhibit the release of excitatory neurotransmitters, thereby achieving analgesic effects [17]. Gabapentin is widely used for TN treatment. It can act on peripheral nerve nociceptors, the spinal conduction pain pathway, and the cerebral cortex [4]. During the occurrence and progression of TN, nerve demyelination damages intraneural mast cells, macrophages, and vascular endothelial cells, leading to inflammatory responses [4]. Gabapentin reduces tumor necrosis factor-α and interleukin-6 levels, which promote inflammation and produce acute response proteins [4]. The dose is started at 300 mg daily and gradually increased by 300 mg every 2–3 days as tolerated, with a maximum dose of 1800 mg daily [17]. Gabapentin has the advantages of fast titration, no drug interactions, no skin reactions, and few adverse effects (e.g., mild somnolence, dizziness, headache, nausea, and edema) [17]. In a meta-analysis of 18 studies involving 1604 patients, gabapentin was found to be superior to carbamazepine in terms of efficacy and safety for primary TN [4]. Gabapentin can be used as an alternative when first-line medications are ineffective or have severe adverse effects [4].

#### 3.4.2. Pregabalin

Pregabalin is a GABA analog that is structurally related to gabapentin and has improved pharmacokinetic properties [14,17]. Pregabalin interacts with the alpha-2-delta subunit of voltage-gated calcium channels [17]. In one study, patients with TN received 150–600 mg of pregabalin daily and were followed up for 1 year, and pain reduction was shown in more than 50–74% of patients [14]. Adverse effects include dizziness, somnolence, peripheral edema, weight gain, headache, and dry mouth [29].

### 3.5. Analgesics

Immediate pain relief is required for the sudden and severe exacerbation of TN pain. Recent recommendations include the use of local anesthetics, mainly lidocaine (ophthalmic, nasal or oral mucosa, trigger point injection, intravenous infusion, and nerve block), anticonvulsants (phenytoin or its prodrug, fosphenytoin), and serotonin agonists (subcutaneous or nasal sumatriptan) [6]. Higher systemic doses of local anesthetics exert neuromodulatory effects by reducing C-fiber transduction of pain signals and inhibiting ectopic discharges from damaged neurons without affecting normal sensory functions [6]. Topical ophthalmic anesthetics (e.g., procaine) relieved pain in some cases [12]. Phenytoin is a voltage-gated sodium channel antagonist [6]. Sumatriptan may exert analgesic effects on TN by reducing pain transmission in the pons [6] and reducing the mechanical compression of the trigeminal nerve root through the vascular loop as a vasoconstrictor [6]. Extremely limited evidence has been reported for NMDA receptor antagonists (magnesium sulfate infusion) [6].

## 4. Interventional Treatments

**Figure 4:**
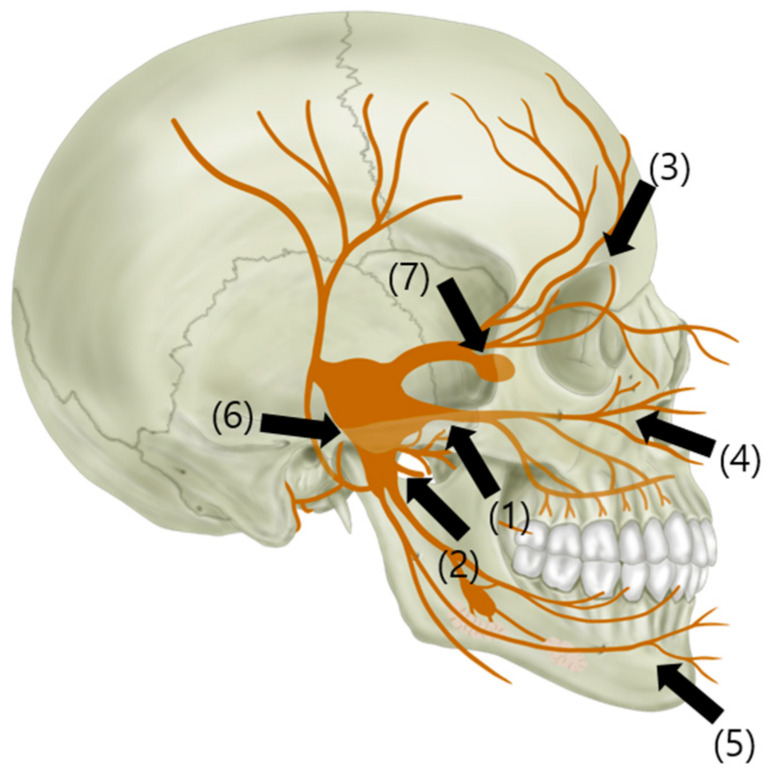
Interventional treatments. (1) Maxillary nerve block; (2) mandibular nerve block; (3) supraorbital nerve block; (4) infraorbital nerve block; (5) mental nerve block; (6) Gasserian ganglion block; (7) sphenopalatine ganglion block.

A nerve block plays an important role in the diagnosis, prognosis, and treatment of pain by intercepting the vicious cycle of pain, blocking sympathetic nerves, expanding vessels in the lesion area, and improving local blood flow [30]. Peripheral interventions involve blocking or destroying a portion of the trigeminal nerve distal to the Gasserian ganglion [19] (Figure 4). Complications include bleeding, infection, sensory abnormalities, and, rarely, diplopia [31]. Fluoroscopy-guided blocks are standardly performed with an observation of the absence of intravascular contrast spread before injecting local anesthetics [32]. Ultrasound provides real-time images of adjacent tissues, bony structures, and vessels and guides the needle trajectory to the target region without radiation exposure [32,33]. However, obtaining high-quality ultrasound images may be technically difficult in the deep trigeminal nerves. Nevertheless, in one study, a maxillary nerve block via the pterygopalatine fossa was successfully performed using ultrasound [32].

### 4.1. Trigeminal and Peripheral Nerves

#### 4.1.1. Maxillary Nerve

The trigeminal nerve has three major branches, ophthalmic (V1), maxillary (V2), and mandibular (V3). The maxillary nerve innervates the maxilla, nasal cavity, sinuses, palate, and mid-face [33]. The maxillary nerve exits the skull base through the foramen rotundum and divides into branches to the pterygopalatine ganglion and then across the pterygopalatine fossa. It runs through the infraorbital foramen and terminates into the inferior palpebral, nasal, and superior labial branches [34]. The maxillary nerve block approaches include the intraoral, infrazygomatic, or suprazygomatic routes [34]. In one patient with TN, pulsed radiofrequency (PRF) ablation of the maxillary nerve and subsequent intranasal sphenopalatine ganglion blocks improved pain for 2 years [35].

#### 4.1.2. Mandibular Nerve

The third branch, the mandibular nerve, is the largest of the three divisions of the trigeminal nerve. The mandibular nerve innervates the mandible, lower teeth, oral mucosa, anterior two-thirds of the tongue, lower lip, temporomandibular joint, and skin of the temporal region. The mandibular nerve immediately passes caudally to the foramen ovale at the posterior margin of the lateral pterygoid plate [36]. In 11 patients with TN, 13 procedures of conventional RF (CRF) ablation (70–90 °C for 90–180 s) of the mandibular nerve improved pain at 1 and 3 months without complications [36].

#### 4.1.3. Supraorbital Nerve

The supraorbital nerve is a branch of the ophthalmic division of the trigeminal nerve. It emerges from the supraorbital notch, which lies within the medial third of the supraorbital margin, 2–3 cm lateral to the midline [33]. It innervates the upper eyelid, forehead, and the anterior half of the scalp, except for the innervation area of the supratrochlear nerve, which is close to the midline [33]. The supraorbital foramen has variations, such as holes or notches [37]. Xie et al. compared pain and numbness following RF ablation of the supraorbital nerve between the hole and notch types [37]. In the notch type, the supraorbital nerve may have some inner or outer deviation, and an increased risk of shifting the needle point during the procedure induces a lower effective rate owing to incomplete destruction of the nerve [37].

#### 4.1.4. Infraorbital Nerve

The infraorbital nerve is the terminal branch of the maxillary division of the trigeminal nerve and provides sensory innervation to the lower eyelid, nose, and upper lip [33]. It emerges from the infraorbital foramen and is accompanied by infraorbital vessels, approximately at the anterior aspect of the maxillary bone and 1 cm below the midpoint of the infraorbital margin [33]. In three patients with first- or second-division TN, infraorbital nerve blocks with a mixture of 4% tetracaine and 0.5% bupivacaine showed prolonged analgesic effects for more than 3 months [38].

#### 4.1.5. Mental Nerve

The mental nerve is one of the two terminal branches of the inferior alveolar nerve and is rooted in the mandibular division of the trigeminal nerve [33]. It innervates the skin of the chin and lower lip. The mental foramen lies 3 cm lateral to the midline and 1 cm above the lower border of the mandible, between the first and second premolar teeth [33]. In a retrospective case series of nine patients with TN, supraorbital, infraorbital, and mental nerve blocks with local anesthetics showed immediate pain relief of >50%, with seven of nine patients completely pain free or under mild anesthesia, and six of nine patients experiencing lasting pain relief for 1–8 months [39].

#### 4.1.6. Peripheral Nerve Radiofrequency and Ablation

Ablative therapies are defined as procedures for the destruction of the involved neural structure and can be performed at different anatomical segments of the nerve [40]. RF ablation applies radio waves directly to the affected nerve to interrupt the pathological transmission of nociceptive signals [41]. Various complications following RF ablation of the Gasserian ganglion have led to increased attention to the peripheral block and RF ablation of the trigeminal nerve. In a randomized trial, pain relief for up to 3 months between peripheral nerves (the supraorbital, infraorbital, and mental nerve) and RF ablation of the Gasserian ganglion did not differ between groups [42]. In a systemic review and meta-analysis of five studies on RF ablation in idiopathic TN, RF ablation of the peripheral nerves showed a non-significantly higher immediate effect rate and a lower association with complications. RF ablation of the Gasserian ganglion showed similar pain relief with a lower recurrence rate but was associated with masticatory weakness [43]. The Gasserian ganglion contains cell bodies (pseudomotor neurons), whereas the peripheral nerves contain Schwann cells, which have a higher capacity for repair than cell bodies. Therefore, the pathway damaged by RF ablation of the peripheral nerves can be repaired and reformed to transmit pain, leading to recurrence [43].

### 4.2. Gasserian Ganglion

#### 4.2.1. Gasserian Ganglion Block

The Gasserian (trigeminal) ganglion is the largest cranial sensory ganglion located within the Meckel’s cave, a cerebrospinal fluid (CSF) filled cavity formed by the dura mater in the middle cranial fossa [32,44]. The trigeminal nucleus is located in the brain stem, which is the aggregation area of the secondary neurons of the trigeminal nerve, whereas the Gasserian ganglion is the gathering area of first-level trigeminal neurons [44]. The Gasserian ganglion is an external structure of the CNS system that is separated from the brain tissue [44]. It contains sensory and motor neurons that receive sensory and motor information from the three branches of the trigeminal nerve [44]. These branches exit the skull through three foramina (superior orbital fissure, foramen rotundum, and foramen ovale) [32]. The Gasserian ganglion is the most commonly used target for the diagnosis and treatment of TN [44]. The first technique for accessing the Gasserian ganglion was described by Hartel in 1910, and the concept of RF ablation was introduced in 1975 [43]. Gasserian ganglion blocks with local anesthetics are used as prognostic blocks before subjecting patients to chemical neurolysis or RF ablation. Bradycardia occurs in some cases when the foramen ovale is punctured [30]. Facial numbness, swelling, ecchymoma, and hematoma at the puncture sites have also been reported [30].

#### 4.2.2. Gasserian Ganglion Radiofrequency Ablation

RF ablation at the Gasserian ganglion selectively destroys pain fibers by thermocoagulation, which helps reduce pain and prevent triggering but may cause dysesthesia [45]. For safe and successful RF ablation of the Gasserian ganglion, the triangular plexus, which comprises the posterior margin of the Gasserian ganglion to the path over the upper petrous ridge, is the best location for generating more selective lesions [46]. In 13 studies with 1146 patients, RF ablation in TN had a success rate of 89.2% and a recurrence rate of 7.9% [41]. CRF generates a 5 to 15 mm electric field that increases the temperature of the affected tissue to over 45 °C, which produces local tissue damage and loss of myelinated nerve fibers. PRF is performed with 20 ms pulses every 0.5 s, allowing time for dissipation of heat and energy, therefore, not exceeding the target temperature over 45 °C [35]. CRF causes structural damage to myelinated and unmyelinated fibers. PRF causes less damage to the surrounding structures and is considered a safe technique, but the results vary [45]. Expected complications include pain recurrence, diminished corneal sensation, masseter weakness, dysesthesia, anesthesia dolorosa, keratitis, transient palsy of cranial nerves III and IV, CSF leakage, corticocavernous fistula, and aseptic meningitis [45]. Elawamy et al. compared the efficacy of continuous RF, PRF, and a combination of both in treating TN [45]. The combination therapy group showed the best pain relief for 6, 12, and 24 months (95%, 85%, and 70%, respectively), followed by the continuous RF and PRF group [45]. The effectiveness of RF versus other percutaneous strategies was reviewed, and RF was found to be superior to glycerol rhizotomy for immediate pain relief; however, RF yielded an excess risk of pain recurrence compared to microvascular decompression [47]. The most common cause of TN pain is neurovascular compression; therefore, pain can be directly resolved by microvascular decompression, as opposed to that by RF [47]. RF ablation of the Gasserian ganglion offers the highest rate of complete pain relief in patients with high surgical risk [42]. In one study, single RF ablation of the Gasserian ganglion showed complete pain relief in 57.7% of patients at 60 months and in 42.2% at 180 months [43]. However, the evidence of safety is insufficient owing to the requirement of experienced technical skills and the use of neurodestructive modalities.

### 4.3. Sphenopalatine Ganglion Block and Radiofrequency Ablation

The sphenopalatine ganglion (SPG) is also known as the pterygopalatine, nasal, or Meckel’s ganglion [48]. It is the largest parasympathetic ganglion associated with branches of the maxillary nerve close to the sphenopalatine foramen [48]. Owing to its proximity to multiple neuroanatomic structures involved in pain perception, the SPG is a target for treating headache and facial pain via blocks, RF ablation, and neurostimulation [48]. As early as 1925, Ruskin described that the SPG was involved in TN pathogenesis and that SPG block relieved TN [35]. Communication between the SPG and maxillary nerve has been described as a mechanism for trigeminal pain in the pathophysiology of cluster headache [49]. The SPG block acts by blocking parasympathetic flow to the cerebral vasculature, allowing cerebral vessels to return to their normal diameter, which relieves headache [48]. The SPG block techniques include the intranasal application of local anesthetics, intraoral approach, and infrazygomatic approach [35,48]. The SPG is located immediately posterior to the middle nasal turbinate. Therefore, it is the only ganglion that can be accessed externally via the nasal mucosa [50]. The intranasal approach is technically simple, has a short procedure duration, and is associated with a low risk of procedure-related complications. These analgesic effects are due to the delivery of local anesthetics, possibly via the maxillary artery plexus surrounding the sphenopalatine foramen [50]. In one study, intranasal spraying of 8% lidocaine decreased paroxysmal TN pain for 4.3 h [48]. The infrazygomatic approach is commonly used for the direct administration of local anesthetics to the SPG and subsequent RF ablation of the SPG. The efficacy of RF ablation of the SPG has mainly been reported for cluster headache and migraine [48]. In a study of 27 patients with various forms of headache and facial pain, including atypical TN, pain was completely relieved in 35% of the cases after RF ablation of the SPG [51].

### 4.4. Alcohol

Chemical ablative agents include alcohol, phenol, and glycerol [52]. They disrupt the transmission of pain signals for 3–6 months by causing Wallerian degeneration distal to the lesion [52]. Alcohol neurolysis has been recommended for intractable visceral cancer pain in the celiac plexus and superior hypogastric or ganglion impar blocks. Potential complications include skin necrosis, neuritis, anesthesia dolorosa, and prolonged motor paralysis [52]. In 465 patients with TN, trigeminal nerve block with alcohol provided immediate complete pain relief in the first block, and the probabilities of remaining pain relief for 1, 2, 3, and 5 years after the procedures were 86.2%, 65.5%, 52.5%, and 33.4%, respectively [53]. The complications are local symptoms, including non-neuralgic pain, a burning sensation, trismus, local infection, and expected loss of sensation along the branch involved in TN, with an incidence of 0.73–3.0% [47]. However, the evidence is scarce because the literature comprises retrospective studies, and there are no randomized controlled trials.

### 4.5. Botulinum Toxin

Botulinum toxin A (BTX-A) injections are a novel treatment option for TN [54,55,56,57,58,59,60]. BTX-A is a neurotoxin derived from the Gram-positive, rod-shaped, spore-forming anaerobic bacterium, *Clostridium botulinum* [57,61]. BTX-A inhibits acetylcholine release at the neuromuscular junction, inhibits the release of inflammatory mediators, and enhances the release of anti-nociceptive neuropeptides (glutamate, substance P, and calcitonin gene-related peptide), which may reduce central and peripheral sensitization [54,62]. BTX-A has an analgesic effect independent of muscle relaxation, possibly by counteracting central sensitization [57,58]. BTX-A has been used to treat muscle hyperactivity and various neurological conditions (migraine, complex regional pain syndrome, diabetic neuropathy, occipital neuralgia, postherpetic neuralgia, and TN) [17,60,63,64]. In an animal neuropathy model, unilateral peripheral administration of BTX-A showed a bilateral therapeutic effect, and BTX-A was localized in the neurons of the bilateral trigeminal ganglion, suggesting that axonal and hematogenous transport of BTX-A may be involved in the therapeutic mechanism [60]. In clinical studies, the injection routes are subcutaneous, intradermal, submucosal, or trigger zones [59,64,65]. Intradermal injection is recommended because of its proximity to the papillary dermis, where unmyelinated sensory nerve endings are located [64]. Transient facial asymmetry and weakness were noted in one study due to the proximity to the facial muscles through the inhibition of acetylcholine release at the neuromuscular junction [64]. The injection point was the facial area with pain, hyperesthesia, and allodynia, which could be mapped with a touch of cotton and a pinprick [64]. The starting dose is 2.5 units in 0.1 cc/cm^2^ [64]. If a patient does not have significant pain relief (>50% pain reduction) after 4 weeks, a booster dose of 2.5–5.0 units/cm^2^ is administered [64]. In a randomized study of classical TN, the epidermal, dermal, and oral submucosal injections of BTX-A showed 70.4% (25 units) and 86.2% (75 units) efficacy, respectively, compared with placebo (32.1%) after 8 weeks [61]. The most commonly used dose of BTX-A is 20–75 units. However, in a pilot study with 13 patients with TN, a minimal dose of 6–9 units of BTX-A was injected transcutaneously (subdermal), and significant pain improvement of over 50% was observed within 10 days after injection, persisting for 60 days [63]. In eight patients with refractory idiopathic TN, a large bolus dose of 100 units of BTX-A was injected into the zygomatic arch region using the blind technique, and the incidence and severity of pain were significantly reduced during the following 6 months, except for mild facial weakness in one case [56]. Using a mixture of botulinum toxin and lidocaine as trigger points for managing acute pain attacks prolongs the effect of lidocaine [6]. In the SPG block, 50 units of botulinum toxin were injected into 10 patients with second-division TN who did not respond to submucosal botulinum toxin injection, resulting in reduced pain scores and frequency at 4 weeks [66]. In a single case of TN, 20 units of BTX-A were injected intraorally into the mental foramen, resulting in reduced pain 6 weeks, 10 weeks, and 5 months after the first, second, and third injections, respectively [67]. In 27 patients with classical TN, 100 units of BTX-A was injected into the maxillary nerve around the pterygopalatine ganglion (50 units) and the mandibular nerve around the Gasserian ganglion (50 units) via the blind technique [68]. The pain intensity and attack frequency were reduced in the first week, second month, and sixth month [68]. However, evidence for a Gasserian ganglion block with BTX-A is absent, and the optimal dose and injection interval evidence is weak for peripheral injections (Table A2, Appendix B).

## 5. Non-Surgical vs. Surgical Treatments

The European Academy of Neurology guidelines recommend that medical management with adequate doses and regular monitoring is needed before considering surgery [69]. However, the optimal number of drugs before surgical referral has not been identified [70]. If pain is not sufficiently controlled medically or if medical treatments are poorly tolerated, microvascular decompression (MVD) is recommended as first-line surgery in classical TN [69]. In a study of 5149 patients, MVD resulted in 62–89% of patients being pain-free at 3–10.9 years of follow-up [69]. However, MVD can be associated with stroke, CSF leaks, and a less than 5% risk of ipsilateral hearing loss [69,70]. For idiopathic TN, MVD and ablative procedures are equal first choices when there is a neurovascular contact; ablative treatments are only recommended when there is no neurovascular contact [69]. Stereotactic radiosurgery, such as gamma knife (GKS), aims a focused beam of radiation at the trigeminal root entry zone [69]. In one study, pain-free results after ablative treatments were 30–66% at 3.1–5.6 years of follow-up in GKS and 26–82% at 3–9.3 years of follow-up in RF thermocoagulation [69]. The most common complications for ablative treatments are facial hypoesthesia, corneal hypoesthesia, and trigeminal motor weakness [69].

## Data Availability

Not applicable.

## Appendix A

**Table A1:** Pharmacotherapy.

	Starting Dose	Dose Titration	Total Dose	Tapering	References
First line					
Carbamazepine	100–200 mg	100–200 mg/2–3 days	1200–1800 mg	200 mg every 7 days	[17,19,20]
Oxcarbazepine	300 mg	300–600 mg/7 days	1200–2400 mg	300 mg every 7 days	[15,19,22]
Second line					
Lamotrigine	25 mg	25–50 mg/week	400–600 mg	50 mg every 7 days	[16]
Baclofen (oral)	5–10 mg	5 mg/3 days	60–80 mg	15 mg every 7 days	[12,23,24]
Third line					
Gabapentin	300 mg	300 mg/2–3 days	900–3600 mg	300 mg every 3 days	[4,17]
Pregabalin	150 mg	50 mg/2–3 days	300–600 mg	100 mg every 7 days	[17]

## Appendix B

**Table A2:** Botulinum toxin.

Administration Route	Patient Number	Total Dose	Effect Duration	References
Intradermal and/or submucosal	40	75 units	12 weeks	[71]
Epidermis, dermis, submucosal	84	25–75 units	8 weeks	[61]
Subcutaneous	11	30–50 units	4 months	[72]
Subcutaneous	88	25–170 units	14 months	[59]
Into the zygomatic arch region	8	100 units	6 months	[56]
Trigger zones	15	50 units	6 months	[65]
Maxillary and mandibular nerve	27	100 units	6 months	[68]
Sphenopalatine ganglion	10	50 units	4 weeks	[66]
